# Videoconferencing-Based Treatment of Alcohol Use Disorders: Analyses of Nonparticipation

**DOI:** 10.2196/formative.6715

**Published:** 2017-09-28

**Authors:** Kristine Tarp, Anna Mejldal, Anette Søgaard Nielsen

**Affiliations:** ^1^ Unit of Clinical Alcohol Research Department of Clinical Research University of Southern Denmark Odense C Denmark

**Keywords:** nonparticipation, refusal to participate, barriers, treatment of alcohol use disorders, alcoholism, treatment refusal, videoconferencing, effectiveness, treatment outcome

## Abstract

**Background:**

We recently conducted a small randomized controlled trial (RCT) aiming to examine the effectiveness of videoconferencing-based treatment of alcohol use disorders in a real-life setting. The patient and participation rates were lower than anticipated.

**Objective:**

The objectives of our study were (1) to examine differences between participants and nonparticipants, and (2) to examine the characteristics of nonparticipants and their reported reasons for not participating.

**Methods:**

First, we analyzed nonparticipation through a comparative analysis of participants and nonparticipants using data from a clinical database, covering all patients starting treatment at the clinic. Second, on the basis of data from an anonymous questionnaire filled out by nonparticipants, we analyzed barriers to participating and the descriptive sociodemographics of nonparticipants who reported technical barriers versus those who did not.

**Results:**

Of 128 consecutive patients starting treatment during the study period, we found no significant differences between participants (n=71) and nonparticipants (n=51) according to sociodemographics, alcohol measures, and composite scores. Of 51 nonparticipants, 43 filled out the questionnaire with reasons for not participating. We derived 2 categories of barriers from the questionnaire: scientific barriers, which were barriers to the scientific study in general (n=6), and technical barriers, which were barriers to using a laptop or videoconferencing specifically (n=27). We found no significant differences in sociodemographics between nonparticipants who reported technical barriers to participating in the study and those who did not note technical barriers. A total of 13 patients elaborated on technical barriers, and 9 patients found videoconferencing impersonal, preferred personal contact, and would rather attend face-to-face treatment at the clinic.

**Conclusions:**

Patient barriers to participating in the RCT were mainly concerned with the technology. There were no significant differences between participants and nonparticipants, nor between nonparticipants who noted technical barriers to participating and those who did not. If a similar study is to be conducted or the solution is to be upscaled and implemented, attention should be given to the user friendliness of the technical equipment and the recruitment process, preparing the patients by emphasizing the information given to them about the technical equipment and its advantages.

## Introduction

Previous studies on videoconferencing-based treatment of alcohol use disorders (AUDs) have found videoconferencing to be a feasible, acceptable, and increasingly available and used option to deliver treatment of AUDs in real-world settings. Evidence for the equivalence of videoconferencing and face-to-face treatment is fairly consistent with regard to results, reliability, credibility, session attendance, and attrition [1-8]. However, a rather large proportion of patients still decline using technology for treatment sessions and prefer attending treatment face-to-face [6,9].

None of the studies on videoconferencing-based treatment of AUDs performed so far have, to our knowledge, mentioned rates of accepting or declining to participate in the study—that is, the number of patients who refused to participate during the recruitment process. Instead, they all reported different rates of completion, from 50% up to almost 100%. Frueh et al [5] conducted a feasibility study among 18 men receiving videoconferencing-based AUD treatment. They reported that 14 participants completed the study. This was similar to participation rates of other patients in their program, who had a completion rate of about 85% [5]. Kirkwood et al compared videoconferencing-based versus face-to-face AUD treatment and reported that 26 out of 27 participants completed the study [7]. Staton-Tindall et al [1] conducted a randomized controlled trial (RCT) of motivational enhancement therapy delivered via videoconferencing among 75 rural alcohol users on community supervision. They reported that 12 out of 24 randomly assigned participants completed the study [1]. Baca and Manuel conducted an RCT of motivational interviewing via videoconferencing, telephone, and face-to-face among rural problem drinkers. They reported that 29 out of 30 randomly assigned participants completed the first of 2 sessions [3] and a 3-month follow-up rate of 90% [4].

We recently conducted a small RCT (registered with The Regional Committees on Health Research Ethics for Southern Denmark, S-20110052) aimed at examining the effectiveness of videoconferencing-based treatment of AUDs in a real-life setting *.* Participation was offered to all 128 consecutive patients in the period of recruitment who wished to start AUD treatment at the clinic. However, of these, 51 patients declined to participate. From this situation arose the opportunity and need to examine why these patients declined to participate, as well as their characteristics. We wanted to gain knowledge about whether and how the nonparticipants differed from the participants, and what barriers were at stake when patients declined to participate in the RCT [10].

The objectives of this analysis of nonparticipation were (1) to examine the differences between participants and nonparticipants, and (2) to examine the characteristics of nonparticipants and their reported reasons for not participating. We pursued the objectives through analyses of nonparticipation by (1) a comparative analysis of participants and nonparticipants using data from a clinical database, covering all patients starting treatment at the clinic, and (2) based on data from an anonymous questionnaire filled out by nonparticipants, an analysis of barriers to participating and an analysis of the descriptive sociodemographics of nonparticipants who reported technical barriers versus those who did not.

## Methods

### Setting

The RCT was carried out in a public outpatient alcohol clinic in Odense, Denmark, between September 2012 and October 2013. At the clinic, AUD treatment is carried out by a multidisciplinary team of social workers, nurses, and psychiatrists. The treatment is conducted according to clinical guidelines [11].

### Sampling

**Figure 1:**
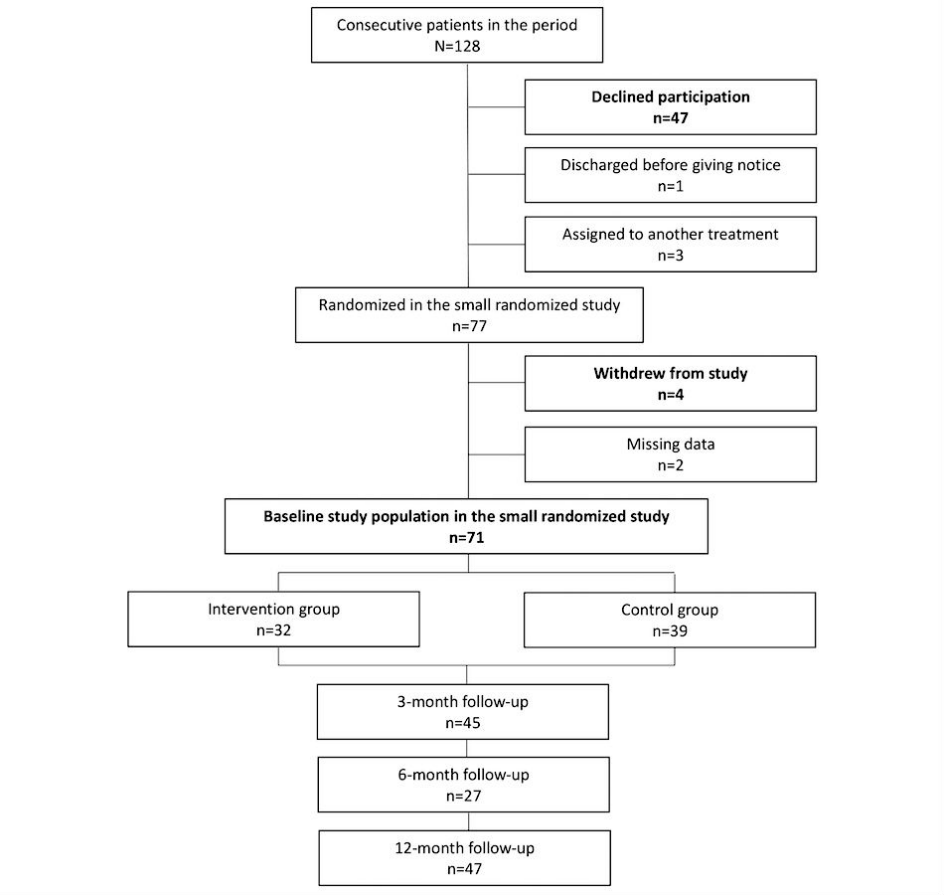
Flowchart of the recruitment process for the small randomized study.

Participants (n=71) consisted of patients who agreed to participate in the RCT. Nonparticipants (n=51) consisted of patients who declined to participate (n=47) or later withdrew from the RCT (n=4). Figure 1 shows the overall recruitment process.

### Data

Data in this study consisted of self-reported data from 2 sources.

The first source was baseline data from a clinical database on participants and nonparticipants. These data were collected by the therapists at the assessment interview at the start of treatment as a part of the normal routine at the clinic. Data were collected by means of the European version of the Addiction Severity Index (EuropASI) [12,13]. The EuropASI provides data on sociodemographics and alcohol measures and collects data on 9 potential problem areas in the patient’s life circumstances: alcohol use, drug use, economic status, employment, legal status, family status, social status, medical status, and psychiatric status. Using EuropASI data, we computed composite scores on the potential problem areas [13]. The composite scores reflect the severity of the 9 potential problem areas during the last month preceding the assessment interview and range from 0 to 1; the higher the score, the greater the severity [12,14]. Studies have demonstrated the Addiction Severity Index (ASI) to be a valid instrument [15,16].

**Figure 2:**
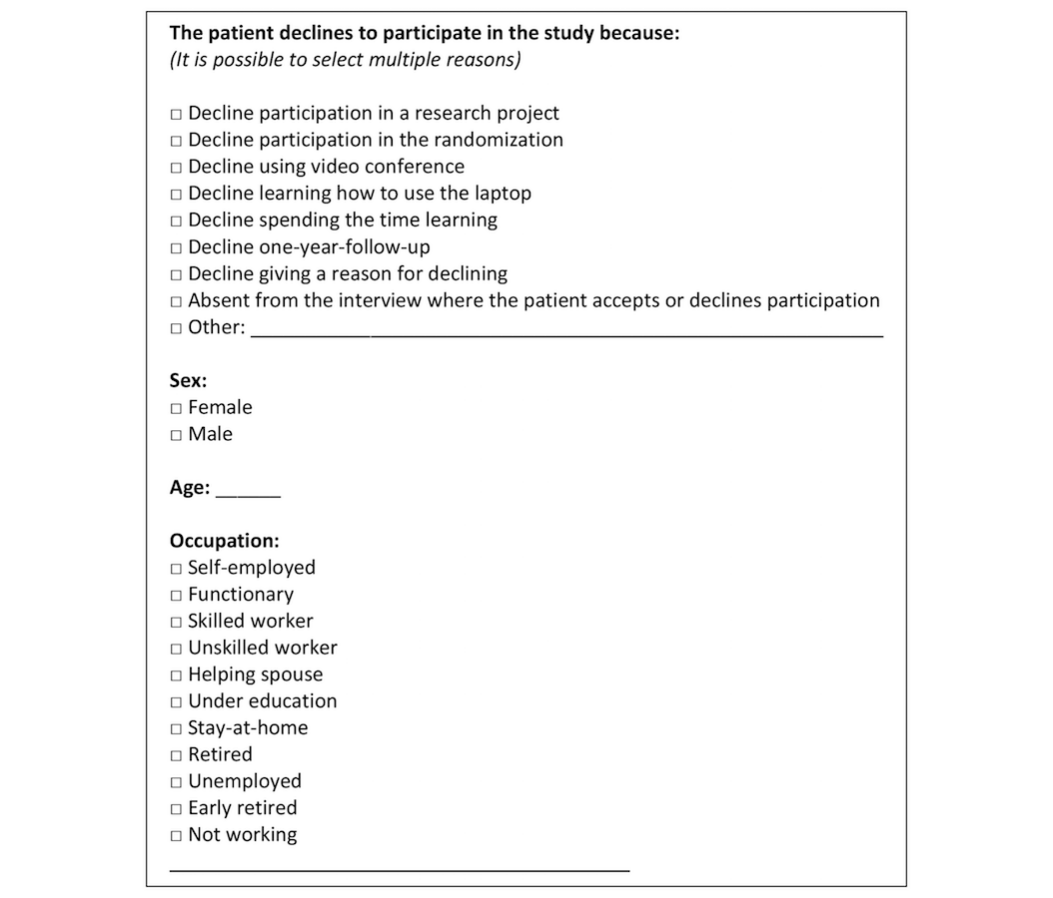
Questionnaire handed out to patients who declined participation in the study.

The second source was data from an anonymous questionnaire. We invited all nonparticipants to fill out the questionnaire at the assessment interview. The questionnaire collected information on nonparticipants’ sex, age, occupation, and reasons for declining to participate. It was possible for patients to state several reasons for refusing participation. It was also possible for patients to decline giving a reason for not participating. Finally, it was possible for patients to elaborate on their answers. Figure 2 shows the questionnaire.

### Statistics

We used Stata v14 (StataCorp LLC) for statistical analyses. To test the relationship between categorical variables, we performed the Pearson chi-square test. If 1 or more of the cells had an expected frequency of 5 or less, we used Fisher exact test. We used the Shapiro-Wilk *W* test for normal data to check for normally distributed data. To compare the means of a normally distributed interval-dependent variable for 2 independent groups, we performed an independent-samples *t* test. When we did not assume the dependent variable to be normally distributed, we used the Wilcoxon-Mann-Whitney test.

## Results

### Participants and Nonparticipants

As Figure 1 shows, we invited 128 patients to participate in the RCT. There were 71 participants and 51 nonparticipants. The nonparticipants consisted of 47 patients who declined to participate and 4 patients who later withdrew from the RCT. Almost all of them had computers of their own, and some of them were accustomed with using videoconferencing.

Table 1 shows the baseline characteristics at treatment start for participants and nonparticipants. We found no significant differences between participants and nonparticipants.

### Barriers to Participating

Of the 51 nonparticipants, 43 filled out the questionnaire describing reasons for declining to participate. From the questionnaire, we derived 2 categories of barriers: scientific barriers, consisting of reasons for deciding against participating in a scientific study as such, and technical barriers, consisting of reasons for deciding against using a laptop or videoconferencing in particular. Table 2 shows the distribution of barriers to participating.

**Table 1:** Baseline characteristics, by participation group (N=128).

Characteristics		Participants (n=71)	Nonparticipants (n=51)	*P* value
**EuropASI^a^** **sociodemographics**				
	Age in years, mean (SD)	46.7 (12.8)	48.6 (11.2)	.49
	Sex (female), % (n)	27 (19)	33 (17)	.43
	Higher/continuing^b^ education (yes), % (n)	82 (58)	75 (38)	.38
	Employed^c^ (yes), % (n)	44 (31)	38 (18)	.50
	Cohabiting (yes), % (n)	59 (42)	52 (25)	.45
**EuropASI alcohol measures**				
	Age in years at onset of excessive^d^ alcohol use, mean (SD)	31.73 (14.14)	33.29 (14.53)	.50
	Years of excessive alcohol use in life, mean (SD)	14.85 (11.14)	17.39 (15.92)	.40
	Days of alcohol use in past month, mean (SD)	19.34 (10.63)	17.74 (11.60)	.55
	Days of excessive alcohol use in past month, mean (SD)	16.76 (10.97)	16.36 (12.01)	.89
**EuropASI composite scores^d^**				
	Alcohol use, mean (SD)	0.70 (0.21)	0.65 (0.23)	.25
	Drug use, mean (SD)	0.03 (0.10)	0.02 (0.04)	.89
	Economic status, mean (SD)	0.59 (0.45)	0.63 (0.46)	.41
	Employment, mean (SD)	0.41 (0.40)	0.45 (0.40)	.58
	Legal status, mean (SD)	0.02 (0.10)	0.03 (0.10)	>.99
	Family status, mean (SD)	0.17 (0.25)	0.17 (0.25)	.53
	Social status, mean (SD)	0.08 (0.18)	0.08 (0.17)	.36
	Medical status, mean (SD)	0.29 (0.39)	0.31 (0.41)	.67
	Psychiatric status, mean (SD)	0.22 (0.23)	0.22 (0.24)	.96

^a^EuropASI: European version of the Addiction Severity Index.

^b^Some respondents with continuing education attended high school first, some did not.

^c^Not necessarily fulltime.

^d^≥5 units a day in at least 3 days a week during the last 30 days.

^e^EuropASI composite scores vary from 0 (no problem) to 1 (extreme problem) in the 30 days preceding the interview.

**Table 2:** Categories of barriers to participation derived from questionnaires.

Barriers to participating		No. of replies^a^
**Scientific barriers**		
	Participating in a research project	6
	Participating in the randomization	1
	Participating in 1-year follow-up	0
	Total^b^	7
**Technical barriers**		
	Using videoconferencing	22
	Learning how to use the laptop	12
	Spending time learning how to use it	13
	Total^c^	47

^a^It was possible for the patients to give multiple replies; 4 patients noted both scientific and technical reasons.

^b^Based on replies from 6 patients.

^c^Based on replies from 27 patients.

**Table 3:** Descriptive sociodemographics of nonparticipants (n=43), according to whether they reported technical barriers.

Characteristics	Technical barriers (n=27)	Nontechnical barriers (n=16)
Age, mean (SD)	49.2 (10.4)	42.5 (14.0)
Sex (female), % (n)	30 (8)	25 (4)
Employed (yes), % (n)	48 (13)	47 (7)

Table 3 shows a descriptive analysis of nonparticipants who had technical barriers to participating, compared with nonparticipants who noted other barriers to participating. No differences were found.

A total of 13 patients elaborated on the technical barriers with regard to using the laptop and videoconferencing, and 9 patients stated that they found it impersonal, preferred personal contact, and would rather attend treatment face-to-face at the clinic. Some of the barriers noted were as follows:

I think it is nice with a conversation; I want to come here and spend the time this way.

It is negative for the relation. This is not a medical clinic. I think videoconferencing creates a distance.

I think the computer will be a visible sign of the treatment. I cannot cope with this.

I currently live next door to the treatment clinic. I already spend a lot of time at the computer.

I would like to physically leave the house because I have a depression.

I have to come for Antabuse anyway.

## Discussion

Not wanting to participate in research studies has been reported as becoming more and more common [10], especially when studies are performed over the Internet [17].

Our RCT had a high rate of nonparticipation; hence, attention should be brought to reasons for avoiding participation. This study found that the primary barrier to participating was reluctance to receive treatment sessions via videoconferencing, as nonparticipants reported preferring personal contact. This finding is supported by a qualitative study also nested within the RCT [18]. Similar studies have also found participants to favor face-to-face meetings. Ruskin et al asked 15 participants about their preferences: 10 participants preferred face-to-face, none preferred videoconferencing, and 5 were indifferent [6]. In a qualitative study, Finn et al [9] found that participants preferred a personal meeting and generally had a negative attitude toward receiving treatment via telephone or the Internet in general. These forms were seen as pretreatment interventions to assess alcohol use and receive treatment guidance [9]. Expanding on this, such wishes may, however, be outweighed by the advantages of videoconferencing with regard to, for example, easier access and less stigma. Hence, videoconferencing might seem ideal as a barrier-decreasing option for patients in rural areas, as well as a pretreatment solution for people with a hazardous level of alcohol use who would not seek face-to-face treatment at a clinic because they would feel stigmatized [9,19].

Other studies in alcohol research have found it challenging to recruit and maintain patients for treatment and studies [20]. Thus, ideas for improving participation rates have been suggested, including piloting, building trust, conducting outreach, making repeated attempts to reach out and stay in contact, and using mixed modes of data collection [10]. One approach to enhance participation among similar patient groups resulted in a 90% follow-up participation rate. The approach entails hiring staff to pay special attention to the recruitment and follow-up processes [21,22]. Thus, future studies regarding videoconferencing-based treatment may find inspiration in these ideas and, for example, improve the recruitment process by emphasizing the information given to patients about the technical equipment and its advantages, and thereby preparing patients more thoroughly. In the future, however, barriers may decrease automatically due to general improvement of technical equipment and patients becoming more and more accustomed with using technical equipment, in health care situations as well.

### Strengths and Limitations

It is a strength in this study that we were able to obtain some data from nonparticipants at all. When using questionnaires to collect self-reported data, response biases should be considered, since they may have an impact on the validity and reliability of the collected self-reported data [23-27]. However, the use of self-reported data has previously been validated [28,29]. It is also a strength that we used data from different sources, as they may be able to supplement each other. It is a limitation that we were not able to combine the data from nonparticipants concerning reasons for not participating with the data from the clinical database, since the reasons for not participating were given anonymously. Hence, we were not able to describe the 2 groups of nonparticipants (declining to participate for technical versus nontechnical reasons) in more detail. Finally, the small sample size indicates a risk of type 2 error and may have consequences for the inferential conclusions that can be drawn from the results.

### Conclusion

Patients’ barriers to participating were mainly concerned with the technology; participation was declined because the patients refused to receive treatment via videoconferencing.

There were no significant differences between participants and nonparticipants, nor between nonparticipants who had technical barriers to participating and those who did not; the small numbers preclude conclusions on how the groups differed.

If a similar study is to be conducted or the solution is to be upscaled and implemented, attention should be given to the user friendliness of the technical equipment. Also the recruitment process should prepare patients by emphasizing the information given to them about the technical equipment and its advantages.

## Abbreviations

ASI: Addiction Severity Index
AUD: alcohol use disorder
EuropASI: European version of the Addiction Severity Index
RCT: randomized controlled trial
